# Effects of sodium chloride on heat resistance, oxidative susceptibility, motility, biofilm and plaque formation of *Burkholderia pseudomallei*

**DOI:** 10.1002/mbo3.493

**Published:** 2017-06-23

**Authors:** Pornpan Pumirat, Muthita Vanaporn, Usa Boonyuen, Nitaya Indrawattana, Amporn Rungruengkitkun, Narisara Chantratita

**Affiliations:** ^1^ Department of Microbiology and Immunology Faculty of Tropical Medicine Mahidol University Bangkok Thailand; ^2^ Department of Molecular Tropical Medicine and Genetics Faculty of Tropical Medicine Mahidol University Bangkok Thailand; ^3^ Mahidol‐Oxford Tropical Medicine Research Unit Faculty of Tropical Medicine Mahidol University Bangkok Thailand

**Keywords:** *Burkholderia pseudomallei*, melioidosis, salt stress, sodium chloride

## Abstract

*Burkholderia pseudomallei* is an environmental saprophyte and the causative agent of melioidosis, a severe infectious disease prevalent in tropical areas, including southeast Asia and northern Australia. In Thailand, the highest incidence of melioidosis is in the northeast region, where saline soil and water are abundant. We hypothesized that *B. pseudomallei* develops an ability to thrive in saline conditions and gains a selective ecological advantage over other soil‐dwelling microorganisms. However, little is known about how an elevated NaCl concentration affects survival and adaptive changes in this pathogen. In this study, we examined the adaptive changes in six isolates of *B. pseudomallei* after growth in Luria‐Bertani medium containing different concentrations of NaCl at 37°C for 6 hr. The bacteria were then investigated for resistance to heat at 50°C and killing by hydrogen peroxide (H_2_O_2_). In addition, flagellar production, biofilm formation, and the plaque formation efficiency of *B. pseudomallei* after culture in saline conditions were observed. In response to exposure to 150 and 300 mmol L^−1^ NaCl, all *B. pseudomallei* isolates showed significantly increased thermal tolerance, oxidative resistance, and plaque‐forming efficiency. However, NaCl exposure notably decreased the number of *B. pseudomallei* flagella. Taken together, these results provide insight into the adaptations of *B*. *pseudomallei* that might be crucial for survival and persistence in the host and/or endemic environments with high salinity.

## 1 INTRODUCTION

*Burkholderia pseudomallei* is a Gram‐negative pathogenic bacterium responsible for melioidosis in humans and animals. This saprophytic organism is found in soil, stagnant water, and rice paddies. Regions in which melioidosis is endemic include southeast Asia, particularly Thailand, and northern Australia (Cheng & Currie, 2005; Wuthiekanun, Smith, Dance, & White, 1995). Rice farmers are considered a high‐risk group for exposure to *B. pseudomallei* especially during the monsoonal and rainy season when there is a lot of mud and surface water in the rice fields (Chaowagul et al., 1989; Cheng & Currie, 2005; Inglis & Sagripanti, 2006; Wiersinga, van der Poll, White, Day, & Peacock, 2006). Infection mainly occurs by inoculation through skin abrasions or inhalation. The clinical features of melioidosis vary considerably, ranging from acute fulminant septicemia to chronic localized infection. In its acute form, death can occur within days of the onset of symptoms. However, the longest reported incubation period between initial acquisition of the organism and subsequent infection is a remarkable 62 years. Furthermore, a high rate of relapse has been recognized (Ngauy, Lemeshev, Sadkowski, & Crawford, 2005). Unfortunately, there is currently no effective vaccine available for the prevention of melioidosis. The treatment of melioidosis generally involves the antibiotics ceftazidime or carbapenem as *B. pseudomallei* exhibits resistance to several empiric antimicrobial therapies.

In Thailand, the highest prevalence of *B. pseudomallei* and the highest incidence of melioidosis are in the northeast region, where saline soil and water are plentiful. The electrical conductivity of soil samples from northeast Thailand ranges from 4 to 100 dS/m, which is higher than that of normal soil from other regions (approximately 2 dS/m) (Development Department of Thailand). We hypothesized that *B. pseudomallei* may develop an ability to adapt to saline conditions and gain cross‐protection to other stress conditions. There is evidence of a link between high NaCl concentrations and an ability to survive in saline conditions in other closely related organisms, namely, the *Burkholderia cepacia* complex (BCC). These organisms are opportunistic pathogens of cystic fibrosis (CF) sufferers (Mahenthiralingam, Baldwin, & Vandamme, 2002; Vandamme et al., 1997) whose lung airways have an increased concentration of NaCl in the surface liquid (Widdicombe, 2001), approximately twofold higher than that of healthy lungs (Joris, Dab, & Quinton, 1993). The potential pathogenic role of *B. pseudomallei* in CF lung disease has also been reported (O'Carroll et al., 2003).

Several studies have shown that exposure to NaCl can influence the adaptive survival and virulence of pathogenic bacteria. The relevance of this has been shown in *Salmonella enterica* serovar Typhimurium (12)*, Staphylococcus aureus* (Park et al., 2012), and *Listeria monocytogenes* (Garner, James, Callahan, Wiedmann, & Boor, 2006), whereby bacteria cultured in medium‐containing high NaCl show increased heat tolerance (Park et al., 2012; Yoon, Park, Oh, Choi, & Yoon, 2013), antibiotic resistance (Yoon et al., 2013), and invasion ability into host cells (Garner et al., 2006; Yoon et al., 2013). Our previous study also showed that *B. pseudomallei* grown under salt stress displayed significantly greater resistance to the antibiotic ceftazidime (Pumirat et al., 2009). Salt‐treated *B. pseudomallei* exhibited greater invasion efficiency into the lung epithelial cell line A549 (Pumirat et al., 2010). However, only one *B. pseudomallei* isolate was used in our previous study and adaptive responses of *B. pseudomallei* to high NaCl concentrations remain largely unknown.

In this study, we further investigated the adaptive response of six *B. pseudomallei* isolates grown in Luria–Bertani (LB) medium with different concentrations of NaCl for 6 hr at 37°C. The concentrations of NaCl used were 0, 150, and 300 mmol L^−1^ which are equivalent to 0, 15, and 30 dS/m, respectively. The bacteria under salt stress were then tested for heat resistance, oxidative susceptibility, swarm motility, flagellar production, and biofilm and plaque formation.

## 2 METHODS

### 2.1 Bacterial strains, growth, and salt treatment

Experiments were performed using six clinical isolates of *B. pseudomallei*: strains 153, 576, 1026b, 1530, 1634, and the reference strain K96243. All strains were obtained from clinical specimens of six patients presenting with melioidosis in northeast Thailand. The bacteria were generally maintained on LB agar at 37°C. To examine the effect of NaCl, *B. pseudomallei* was subcultured in NaCl‐free LB broth and incubated at 37°C with shaking at 200 rpm overnight. The bacteria were then inoculated at a dilution of 1:10 into 10 ml of LB broth containing 0, 150, and 300 mmol L^−1^ NaCl and incubated at 37°C for 6 hr with shaking. The salt‐treated and untreated *B. pseudomallei* were adjusted to an OD_600_ of 0.15. A serial dilution was performed to determine the number of colony‐forming units (CFU) to obtain the starting number of bacteria.

### 2.2 Heat resistance assay

A heat stress resistance assay was performed as described previously (Vanaporn, Vattanaviboon, Thongboonkerd, & Korbsrisate, 2008) with some modifications. Briefly, *B. pseudomallei* cultured in LB medium containing different salt concentrations (0, 150, and 300 mmol L^−1^ NaCl) at 37°C for 6 hr were washed with phosphate‐buffered saline (PBS) and resuspended in PBS to an OD_600_ of 0.15. One milliliter of the bacterial suspension was then added into a prewarmed tube and incubated at 50°C for 15 min. Before and after heat challenge, bacterial survival was enumerated on LB agar plates after incubating at 37°C for 24 hr. The number of surviving bacteria was expressed as a percentage of the viable cells.

% Survival = CFU (heat exposure) × 100/CFU (without heat exposure)

### 2.3 Oxidative stress assay

The survival of *B. pseudomallei* under oxidative conditions was determined by observing the number of viable bacteria after exposure to an oxidative agent. After 6 hr of culturing in LB medium containing different salt concentrations (0, 150, and 300 mmol L^−1^ NaCl), *B. pseudomallei* cells were harvested, washed, and resuspended in PBS. The bacterial concentration was adjusted to an OD_600_ of 0.15. Then, 100 μl of bacterial suspension was treated with H_2_O_2_ (at a final concentration of 1 μmol L^−1^) or left untreated at room temperature for 15 min. A 10‐fold dilution of treated and untreated bacteria was performed and plated on LB agar. After incubation at 37°C for 24 hr, colonies were counted. The number of colonies of treated bacteria was compared with that of untreated bacteria (without oxidant) and presented as the % bacterial survival.

% Survival = CFU (with oxidant) × 100/ CFU (without oxidant)

### 2.4 Motility assay

A motility assay was undertaken using the swarm plate method as previously described (Deziel, Comeau, & Villemur, 2001). Briefly, *B. pseudomallei* were grown in LB broth with 0, 150, or 300 mmol L^−1^ NaCl for 6 hr at 37°C. Bacterial pellets were collected, washed, and adjusted in PBS to approximately 10^8^ CFU/ml. Swarm plates were inoculated by placing 2 μl of the prepared inoculum onto the agar surface at the center of the plate. The diameter of the swarming motility zone was measured from the point of inoculation after incubation at 37°C for 24 hr.

### 2.5 Electron microscopic examination

The presence of *B. pseudomallei* flagella was examined using a transmission electron microscope. Fifty microliters of *B. pseudomallei* grown in LB broth with different salt concentrations was harvested and dropped onto parafilm. Formvar‐coated carbon grids were placed on top of the parafilm for 10 min to transfer the bacterial cells. The liquid was then carefully removed with filter paper. The samples were stained with 1% uranyl acetate for 10 min, then the liquid was removed again. The grid was dried at room temperature overnight. Bacteria were observed under a Hitachi Electron Microscope H‐7000 (Japan). The presence of bacterial flagella was recorded for 100 bacteria per condition.

### 2.6 RNA preparation and real‐time RT‐PCR

RNA was isolated from 6 hr culture of *B. pseudomallei* grown at 37°C by adding 10 ml of RNAprotect bacterial reagent (QIAGEN) to 5 ml of bacteria culture and incubating for 5 min at room temperature. Subsequently, total RNA was extracted from bacterial pellets using Trizol (Invitrogen, Carlsbad, CA, USA) according to the manufacturer's instructions and treated with DNase (NEB, MA, USA) for 10 min at 37°C before use. Conventional PCR for 23S RNA gene was used to verify that there was no gDNA contamination in the DNase‐treated RNA samples. Real‐time RT‐PCR was performed for six genes (*rpoE*,* groEL*,* htpG bopA*,* bopE,* and *bipD)* using Brilliant II SYBR^®^ Green QPCR Master Mix, one step (Agilent Technologies, Santa Clara, CA, USA) with following conditions: reverse transcription at 50°C for 30 min, enzyme activation at 95°C for 10 min, then 40 cycles of denaturation at 95°C for 30 s, annealing at 55°C for 1 min, and melting curve analysis at 72°C for 1 min in a CFX96 Touch^™^ Real‐Time PCR Detection System (CA, USA). Real‐time RT‐PCR primers are listed in Table 1. Relative mRNA levels were determined by fold change in expression, calculated by 2^−ΔΔCT^ using the relative mRNA level of 23S RNA, representing a house‐keeping gene expression, as a baseline for comparison.

**Table 1:** Oligonucleotide primers used in this study

Primers	Sequences (5′–3′)	Sources
RpoE 36	CTCCAAATACCACCGCAAGAT	(Korbsrisate et al., 2005)
RpoE 37	TATCCCTTAGTTGGTCCG	
Gro1	AGGACGGCGACTTGCTTGT	(Vanaporn et al., 2008)
Gro2	TTCCAAGACCAGTCGACAAC	
Htp1	TACAGCAACAAGGAAATCT	
Htp2	CACTCCTCCTTCTTCATCA	
BopA F	GTATTTCGGTCGTGGGAATG	(Pumirat et al., 2010)
BopA R	GCGATCGAAATGCTCCTTAC	
BopE F	CGGCAAGTCTACGAAGCGA	
BopE R	GCGGCGGTATGTGGCTTC G	
BipD F	GGACTACATCTCGGCCAAAG	
BipD R	ATCAGCTTGTCCGGATTGAT	
23s F	TTTCCCGCTTAGATGCTTT	
23s R	AAAGGTACTCTGGGGATAA	

### 2.7 Biofilm formation assay

Quantification of biofilm formation was performed using a microtiter plate assay as previously described (Leriche & Carpentier, 2000; Stepanovic, Vukovic, Dakic, Savic, & Svabic‐Vlahovic, 2000). Briefly, biofilm formation of *B. pseudomallei* was induced in trypticase soy broth at 37°C for 24 hr. After incubation, the adherent bacteria were washed using deionized water three times and fixed with 99% methanol for 15 min at room temperature. The bacteria were stained for 15 min with 1% crystal violet and solubilized with 33% (v/v) glacial acetic acid. The quantity of biofilm was measured at 630 nm using a microplate reader (Bio‐Rad). Each *B. pseudomallei* isolate was assayed in duplicate, using eight wells per experiment.

### 2.8 Plaque formation assay

Plaque‐forming efficiency was assessed as previously described (Pumirat et al., 2014). HeLa cells were infected with *B. pseudomallei* at a multiplicity of infection of 20 and incubated at 37°C with 5% CO_2_ for 2 hr. Thereafter, the infected cell monolayers were washed and replaced with medium‐containing kanamycin (250 μg/ml). The plates were incubated at 37°C in a humidified 5% CO_2_ atmosphere for 20 hr. Plaques were stained with 1% (w/v) crystal violet in 20% (v/v) methanol and counted by microscopy. Plaque‐forming efficiency was calculated by determining the number of plaques per CFU of bacteria added per well.

### 2.9 Statistical analysis

All assays were conducted in triplicate, and an unpaired *t*‐test of independent experiments was performed using the GraphPad Prism 6 program (STATCON). Results were considered significant at a *p* ≤ .05.

## 3 RESULTS

### 3.1 NaCl stress induces cross‐protection against heat and oxidative agents

Different growth rates may affect the number of viable bacteria under NaCl stress conditions. Therefore, prior to observing the effect of NaCl stress on cross‐protection against heat and oxidative agents, the individual growth of six clinical *B. pseudomallei* isolates (K96243, 153, 576, 1026b, 1530, and 1634) from six patients in northeast Thailand was compared in LB broth containing different NaCl concentrations. Strains K96243, 153, 576, and 1026b were selected as these have been used extensively as reference isolates, and sequence type data are available (K96253, ST10; 153, ST15, 576; ST 501 and 1026b; ST102). Strains 1530 and 1634 were isolated from blood samples of two cases in northeast Thailand and used for comparison. In our previous study, *B*. *pseudomallei* K96243 demonstrated growth impairment during culture in LB containing 470 mmol L^−1^ NaCl (Pumirat et al., 2010). In this study, we investigated the growth kinetics of six *B*. *pseudomallei* isolates in LB media containing 0, 150, or 300 mmol L^−1^ NaCl for 6 hr after incubation at 37°C. Similar growth curves were observed for the six isolates under conditions of 0, 150, and 300 mmol L^−1^ NaCl (Figure S1). Therefore, salt concentrations ranging from 0 to 300 mmol L^−1^ and a culture time of 6 hr were chosen for further investigations.

**Figure 1:**
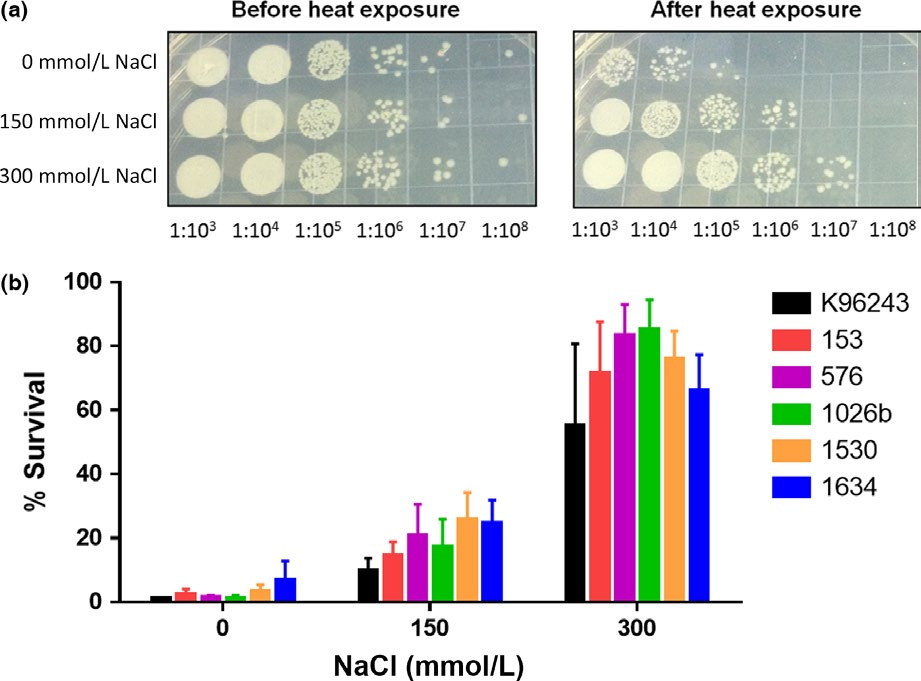
Resistance to heat of *Burkholderia pseudomallei* after growth in Luria–Bertani (LB) broth containing 0, 150, or 300 mmol L^−1^ NaCl. (a) Cell viability of *B. pseudomallei* K96243 before and after heat treatment at 50°C for 15 min. Colony‐forming units were enumerated on LB agar plates after incubation at 37°C for 24 hr. (b) Percent survival of six *B. pseudomallei* isolates after heat treatment at 50°C for 15 min. 100% viability corresponds to the colony‐forming unit count of unexposed bacteria. The data were obtained from at least three experiments. Error bars represent the standard deviation of the mean for experiments performed in triplicate

To evaluate the effect of NaCl on heat resistance in *B*. *pseudomallei*, six *B*. *pseudomallei* isolates were cultured in LB broth with different concentrations of NaCl for 6 hr to reach the log phase of bacterial growth, followed by heating at 50°C for 15 min. Figure 1 shows the percentage of surviving bacteria and demonstrates a significant difference in heat resistance between *B. pseudomallei* isolates cultured in NaCl‐free medium and those cultured in LB with 150 mmol L^−1^ NaCl (*p *=* *.014 for K96243, *p *=* *.011 for 153, *p *=* *.028 for 576, *p *=* *.027 for 1026b, *p *=* *.011 for 1530, and *p *=* *.040 for 1634) or those cultured in LB with 300 mmol L^−1^ NaCl (*p *=* *.020 for K96243, *p *=* *.004 for 153, *p *<* *.001 for 576, *p *<* *.001 for 1026b, *p *<* *.001 for 1530, and *p *=* *.002 for 1634). In addition, the data also showed a significant difference in the percentage of bacterial survival between *B. pseudomallei* isolates cultured in LB supplemented with 150 and 300 mmol L^−1^ NaCl (*p *=* *.038 for K96243, *p *=* *.002 for 153, *p *=* *.001 for 576, *p *<* *.001 for 1026b, *p *=* *.002 for 1530, and *p *=* *.008 for 1634). The mean and standard deviation (SD) of bacterial survival in NaCl‐free medium of the six *B. pseudomallei* isolates after heat treatment were 2.2 ± 0.5%. By contrast, the mean and SDs of bacterial survival of the six isolates in medium containing 150 mmol L^−1^ and 300 mmol L^−1^ NaCl were 18.2 ± 2.9% and 67.9 ± 8.9%, respectively. These data clearly revealed that salinity is associated with increased resistance of *B. pseudomallei* to heat stress.

**Figure 2:**
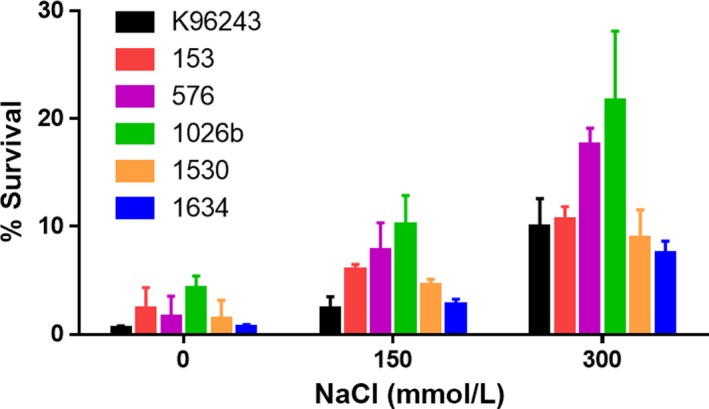
Susceptibility to oxidative stress of six *Burkholderia pseudomallei* isolates grown in Luria–Bertani (LB) broth containing 0, 150, and 300 mmol L^−1^ NaCl. Susceptibility to killing by 1 μmol L^−1^ H_2_O_2_ was determined at 15 min. Surviving bacteria were enumerated on LB agar plates after incubation at 37°C for 24 hr and were expressed as the % survival. The data were obtained from three experiments. Error bars represent the standard deviation of the mean for three experiments

Activation of the oxidative response during survival in salt stress has been reported for various bacteria (den Besten, Mols, Moezelaar, Zwietering, & Abee, 2009; Metris, George, Mulholland, Carter, & Baranyi, 2014). We investigated the effect of NaCl on oxidative susceptibility of six *B*. *pseudomallei* isolates grown in different NaCl concentrations. Equal numbers of salt‐treated and untreated *B. pseudomallei* were exposed to 1 μmol L^−1^ H_2_O_2_ for 15 min, and their survival on LB agar was determined (Figure 2). The percentage of surviving bacteria among the *B. pseudomallei* isolates grown in salt‐free medium in the presence of H_2_O_2_ was significantly lower than the bacteria exposed to salt at a concentration of 150 mmol L^−1^ NaCl (*p *=* *.046 for K96243, *p *=* *.039 for 153, *p *=* *.019 for 576, *p *=* *.027 for 1026b, *p *=* *.043 for 1530, and *p *=* *.014 for 1634), or those exposed to 300 mmol L^−1^ NaCl (*p *=* *.004 for K96243, *p *=* *.004 for 153, *p *<* *.001 for 576, *p *=* *.010 for 1026b, *p *=* *.011 for 1530, and *p *<* *.001 for 1634). These data also showed a significant difference in the percentage of bacterial survival between *B. pseudomallei* isolates cultured in LB medium supplemented with 150 mmol L^−1^ and 300 mmol L^−1^ NaCl under oxidative stress conditions (*p *=* *.010 for K96243, *p *=* *.004 for 153, *p *=* *.005 for 576, *p *=* *.046 for 1026b, *p *=* *.049 for 1530, and *p *<* *.001 for 1634). In the presence of H_2_O_2,_ the mean survival rate of untreated *B*. *pseudomallei* isolates was 1.7 ± 0.6%, compared with 5.6 ± 1.2% for those exposed to 150 mmol L^−1^ NaCl and 12.7 ± 2.3% for those exposed to 300 mmol L^−1^ NaCl. These data indicated that preexposing bacteria to salt stress reduced susceptibility to H_2_O_2_ in *B*. *pseudomallei*.

**Figure 3:**
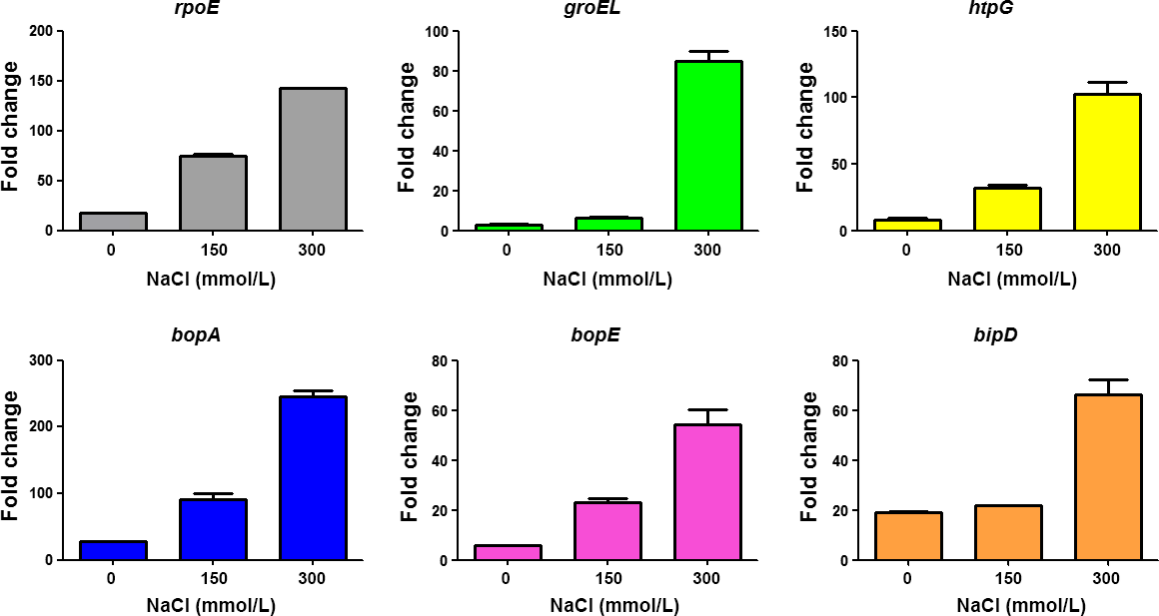
Fold change of *rpoE*,* groEL*,* htpG, bopA, bopE, and bipD* genes in *Burkholderia pseudomallei* K96243 grown in Luria–Bertani (LB) broth containing 0, 150, and 300 mmol L^−1^ NaCl. RNA of *B. pseudomallei* grown in LB broth with different NaCl concentrations for 6 hr was used for determination of gene expression by quantitative real‐time RT‐PCR using the Brilliant II SYBR ^®^ Green QPCR Master Mix, one step (Agilent Technologies, Santa Clara, CA, USA) according to the manufacturer's recommendation. Relative mRNA levels were determined by fold changes in expression, calculated by 2^−ΔΔCT^. 23S rRNA gene was used for normalization. Error bars represent the standard deviation of the means for experiments performed in triplicate

The response of *B. pseudomallei* to heat and oxidative stress has been reported to be dependent on various cellular components, including transcription factors, heat shock proteins, and virulent proteins (Jitprasutwit et al., 2014; Korbsrisate et al., 2005; Vanaporn et al., 2008). We therefore investigated whether NaCl affects the expression of the *rpoE*,* groEL*,* htpG, bopA*,* bopE,* and *bipD*. The *rpoE*,* groEL*, and *htpG* genes were selected because they code transcription factors or heat shock proteins that have previously been reported to be involved in heat and oxidative stress (Jitprasutwit et al., 2014; Korbsrisate et al., 2005; Vanaporn et al., 2008). The *bopA*,* bopE,* and *bipD* were T3SS genes which may be important for cell invasion (Gong et al., 2011; Muangsombut et al., 2008; Stevens et al., 2003). Real‐time RT‐PCR results showed that *B. pseudomallei* K96243 when exposed to NaCl (150 and 300 mmol L^−1^) exhibited increased expression of all tested genes, compared with bacteria grown under NaCl‐free conditions (Figure 3). These data suggested that NaCl is involved in increasing the expression of stress response proteins, which might be responsible for the enhanced resistance of *B*. *pseudomallei* to heat and oxidative stress.

### 3.2 NaCl decreases the expression of *B. pseudomallei* flagella

Motility is a crucial factor for bacterial pathogenesis. Using a microarray, we previously demonstrated that *B*. *pseudomallei* grown under high NaCl conditions exhibited downregulation of the flagella biosynthesis sigma factor gene “*fliA*” (*bpsl3291*) (Pumirat et al., 2010). Therefore, in this study, we further examined whether salt affects *B*. *pseudomallei* swarm motility. Six isolates of *B*. *pseudomallei* were grown in LB broth containing different concentrations of NaCl (0, 150, or 300 mmol L^−1^) for 6 hr, then equal numbers of bacteria for each isolate were used to inoculate swarm agar medium. After incubation at 37°C for 24 hr, the diameter of the swarming zone was measured (Figure S2). The mean and SDs of the swarming zone diameters of the six *B*. *pseudomallei* isolates were 23.7 ± 0.9, 21.8 ± 1.2, and 17.4 ± 1.6 mmol L^−1^ for bacteria exposed to 0, 150, and 300 mmol L^−1^ NaCl, respectively (Table 2).

**Table 2:** Effect of NaCl on the swarming motility of *B. pseudomallei*

*B. pseudomallei* isolates	Diameter of swarm zone (mmol L^−1^)		
	0 mmol L^−1^ NaCl	150 mmol L^−1^ NaCl	300 mmol L^−1^ NaCl
K96243	24.0 ± 7.0	21.3 ± 6.7	17.7 ± 7.2
153	27.3 ± 4.6	26.3 ± 5.5	23.5 ± 4.4
576	24.7 ± 7.6	24.0 ± 8.2	16.0 ± 9.5
1026b	22.0 ± 7.0	21.7 ± 7.2	19.0 ± 8.2
1530	23.3 ± 9.1	18.3 ± 5.5	16.3 ± 6.4
1634	20.7 ± 2.3	19.3 ± 3.1	11.7 ± 8.1

Data represent the mean ± SD of three experiments each performed in triplicate.

To determine whether altered expression of the *fliA* gene affects bacterial flagella, we examined the number of flagella on the six *B*. *pseudomallei* isolates during growth under different salt conditions using an electron microscope. The results showed that the number of flagella decreased with increasing concentrations of NaCl (Figure S3). The number of flagella counted on 100 bacteria for each of the six isolates is shown in Table 3. The majority of *B*. *pseudomallei* isolates (70.7 ± 3.5%) grown in LB with 300 mmol L^−1^ NaCl showed no flagella. By contrast, only 38.0 ± 3.8% and 49.3 ± 4.3% of *B. pseudomallei* cultured in NaCl‐free and 150 mmol L^−1^ NaCl‐supplemented media, respectively, had no flagella. The number of unflagellated bacteria among the *B*. *pseudomallei* isolates grown in 300 mmol L^−1^ NaCl‐supplemented medium was therefore significantly higher than among those grown in salt‐free (*p *<* *.001) or 150 mmol L^−1^ NaCl‐supplemented medium (*p = *.003, respectively). This phenomenon indicated that salinity affects flagella production in *B. pseudomallei*.

**Table 3:** Effect of NaCl on the number of flagella expressed on *Burkholderia pseudomallei*

*B. pseudomallei* isolates	NaCl (mmol L^−1^)	% Bacteria with flagella		
		0	1–3	>3
K96243	0	36	50	14
	150	52	36	12
	300	76	24	0
153	0	36	52	12
	150	52	28	20
	300	60	40	0
576	0	24	42	24
	150	32	60	8
	300	76	24	0
1026b	0	36	56	8
	150	44	56	0
	300	80	20	0
1530	0	52	44	4
	150	52	44	4
	300	60	40	0
1634	0	44	52	4
	150	64	32	4
	300	72	24	4

Data represent the mean ± SD of three experiments each performed in triplicate. One hundred bacterial cells were counted to determine the number of flagella.

### 3.3 Effect of NaCl on *B. pseudomallei* biofilm formation

*B*. *pseudomallei* can produce biofilm, which may offer protection against hostile conditions such as antibiotic treatment, salinity, and immune responses (Cheng & Currie, 2005; Inglis & Sagripanti, 2006; Kamjumphol, Chareonsudjai, Chareonsudjai, Wongratanacheewin, & Taweechaisupapong, 2013). We therefore tested whether *B*. *pseudomallei* biofilm formation is affected by salt stress. Six isolates of *B*. *pseudomallei* were grown in LB broth with different concentrations of NaCl for 6 hr at 37°C prior to the induction of biofilm formation. The results in Table 4 demonstrate the biofilm formation capacity of each of the *B*. *pseudomallei* isolates. The mean OD values and SDs of the biofilm formation capacity of the *B*. *pseudomallei* isolates increased from 0.19 ± 0.01 to 0.24 ± 0.03 and then to 0.31 ± 0.03 when bacteria were grown in the presence of 0, 150, and 300 mmol L^−1^ NaCl, respectively. Although, each of the *B*. *pseudomallei* isolates tended to show increased biofilm formation when grown in the presence of NaCl compared with those grown in 0 mmol L^−1^ NaCl, we could not detect a significant difference in biofilm formation when comparing bacteria grown in the presence of 0, 150, and 300 mmol L^−1^ NaCl.

**Table 4:** Effect of NaCl on biofilm formation of *Burkholderia pseudomallei*

*B. pseudomallei* isolates	Corrected OD_630_ nm		
	0 mmol L^−1^ NaCl	150 mmol L^−1^ NaCl	300 mmol L^−1^ NaCl
K96243	0.16 ± 0.03	0.21 ± 0.07	0.23 ± 0.07
153	0.24 ± 0.12	0.33 ± 0.18	0.35 ± 0.18
576	0.14 ± 0.01	0.16 ± 0.02	0.22 ± 0.04
1026b	0.20 ± 0.07	0.35 ± 0.22	0.45 ± 0.27
1530	0.17 ± 0.04	0.18 ± 0.04	0.23 ± 0.03
1634	0.21 ± 0.03	0.23 ± 0.03	0.25 ± 0.01

Data represent the mean ± SD of three experiments each performed in triplicate.

### 3.4 NaCl affects *B. pseudomallei* plaque formation

**Figure 4:**
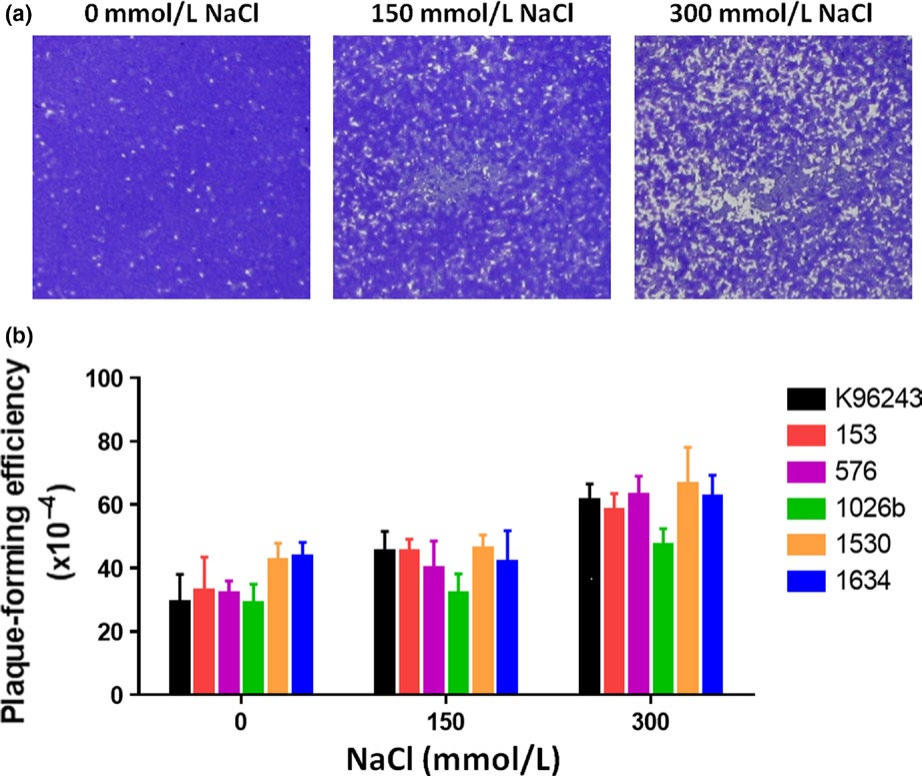
Plaque formation by *Burkholderia pseudomallei* after growth in Luria–Bertani (LB) broth containing 0, 150, and 300 mmol L^−1^ NaCl. (a) Images of plaques formed by *B. pseudomallei* K96243. Representative images of HeLa cell monolayers after infection with *B. pseudomallei* K96243, which had been grown in LB broth containing 0, 150, or 300 mmol L^−1^ NaCl for 20 hr. (b) Plaque‐forming efficiency of six *B. pseudomallei* isolates. HeLa cells were infected with *B. pseudomallei* grown in LB broth containing 0, 150, or 300 mmol L^−1^ NaCl at a multiplicity of infection of 20. The infected cells were stained with crystal violet after 20 hr incubation. Plaque‐forming efficiency was calculated as the number of plaques × 100/number of colony‐forming units of bacteria added per well. Error bars represent the standard deviation of the means for experiments performed in triplicate

*B. pseudomallei* is a facultative intracellular bacteria that harbors the ability for cell‐to‐cell spread (Kespichayawattana, Rattanachetkul, Wanun, Utaisincharoen, & Sirisinha, 2000), which is an important characteristic for pathogenesis. Previously, NaCl was found to increase expression of the *Burkholderia* secretion apparatus (Bsa) type III secretion system (T3SS), which involved a virulence‐associated interaction with the host cell (Pumirat et al., 2010). In particular, the translocon “BipB” and the secreted effector protein “Cif” homolog in *B. pseudomallei* were reported to induce cell‐to‐cell dissemination (Pumirat et al., 2014; Suparak et al., 2005). Hence, we investigated whether salt stress affects cell‐to‐cell spread of *B. pseudomallei*. Six *B. pseudomallei* isolates grown in LB with different concentrations of NaCl for 6 hr at 37°C were assessed for plaque formation. Figure 4a demonstrates plaque formation in the HeLa cell line induced by *B. pseudomallei* K96243 when grown in 0, 150, and 300 mmol L^−1^ NaCl. The mean and SD of the plaque‐forming efficiency of *B. pseudomallei* isolates grown in 300 mmol L^−1^ NaCl were 59.8 ± 2.8, compared with 41.7 ± 2.2 for bacteria grown in 150 mmol L^−1^ NaCl and 35.0 ± 2.7 for those grown in NaCl‐free LB. All *B. pseudomallei* isolates grown in the presence of 300 mmol L^−1^ NaCl showed significantly increased plaque formation relative to bacteria cultured in NaCl‐free medium (*p *=* *.004 for K96243, *p *=* *.021 for 153, *p *=* *.002 for 576, *p *=* *.017 for 1026b, *p *=* *.032 for 1530, and *p *=* *.016 for 1634). Moreover, we also observed a significant difference in the plaque‐forming capacity of all *B. pseudomallei* isolates cultured in LB supplemented with 300 mmol L^−1^ NaCl compared with those cultured in 150 mmol L^−1^ NaCl (*p *=* *.027 for K96243, *p *=* *.029 for 153, *p *=* *.019 for 576, *p *=* *.033 for 1026b, *p *=* *.048 for 1530, and *p *=* *.042 for 1634). This finding indicated the influence of NaCl on *B*. *pseudomallei* pathogenesis.

## 4 DISCUSSION

*B. pseudomallei* is a saprophyte that can survive and multiply under different environmental conditions (Cheng & Currie, 2005; Dharakul & Songsivilai, 1999; White, 2003). It is a difficult microorganism to kill. It can inhabit harsh environments for many years, especially in endemic areas, including northeast Thailand (Wuthiekanun et al., 1995) where saline soil and water are abundant. *B. pseudomallei* was reported as potential opportunist pathogens of CF patients (Mahenthiralingam et al., 2002; O'Carroll et al., 2003; O'Sullivan et al., 2011; Vandamme et al., 1997), who have a high concentration of NaCl in their lung airway surface liquid. Adaptive responses of *Burkholderia* species, including *B. pseudomallei*, to high salt conditions have been investigated previously (Inglis & Sagripanti, 2006; O'Quinn, Wiegand, & Jeddeloh, 2001; Pumirat et al., 2009, 2010), however, the mechanisms underlying these remain poorly understood. This study demonstrated the adaptive phenotypes of six *B. pseudomallei* isolates to NaCl in various concentrations. The concentrations of NaCl used in our experiments were in the range of salt concentrations found in the soil and water in northeast Thailand. We showed that adaptations under salt stress conditions were associated with cross‐protection against other environmental stresses, as well as increased pathogenicity.

Our present study verified that the growth rate of six *B. pseudomallei* isolates in LB containing 0, 150, and 300 mmol L^−1^ NaCl remained constant. We therefore conducted our experiments within this range of concentrations. Although high salinity seems to be a disadvantage for *B. pseudomallei*, as high salt (≥470 mmol L^−1^ NaCl) diminished bacterial growth (Pumirat et al., 2010; Wang‐Ngarm, Chareonsudjai, & Chareonsudjai, 2014), *B. pseudomallei* would regularly encounter a high salinity environment in its physiological habitat. In this study, we demonstrated that NaCl enhanced the ability of *B. pseudomallei* to survive under heat and oxidative stress. Several studies in other bacteria, such as *Bacillus cereus* (den Besten et al., 2009), *Bacillus subtilis* (Volker, Mach, Schmid, & Hecker, 1992), and *Escherichia coli* (Gunasekera, Csonka, & Paliy, 2008), have also reported that activation of the salt stress response conferred cross‐protection against other stresses, that is, increased resistance to heat and H_2_O_2_. Recently, Yuan, Agoston, Lee, Lee, & Yuk, (2012) and Yoon et al., (2013) also showed that the heat resistance of *Salmonella enterica* was increased after exposure to NaCl. Moreover, it is evident that growing *Vibrio harveyi* in LB broth supplemented with 2% NaCl (34.2 mmol L^−1^) resulted in increased resistance to menadione killing compared with the same organism grown in normal LB broth (Vattanaviboon, Panmanee, & Mongkolsuk, 2003). It is possible that the salt stress adaptation may reflect the ability of these bacteria, including *B. pseudomallei*, to survive under hostile environmental conditions, such as high temperature and oxidative stress.

As *B. pseudomallei* is an intracellular organism, it has the capability to survive in phagocytic cells (Allwood, Devenish, Prescott, Adler, & Boyce, 2011). While trafficking within macrophages, *B. pseudomallei* may be exposed to oxidative stress. Interestingly, Scott & Gruenberg (2011) reported that chloride and sodium ion channels play important roles in regulating the phagosomal environment through counter ion regulation and charge compensation of macrophages. Therefore, the salt content in the phagosome may promote bacterial resistance to oxidative stress and allow *B. pseudomallei* to survive within the host cell.

These oxidative and heat protective effects of NaCl could be a result of the increased expression of stress response cellular components. The increased expression of the *rpoE* and *groEL* genes detected in this study was in agreement with previous reports for the *B. pseudomallei* transcriptome (Pumirat et al., 2010) and secretome (Pumirat et al., 2009) under high salinity conditions. The expression of *groEL* (*bpss0477*) and *rpoE* (*bpsl2434*) was upregulated in *B. pseudomallei* cultured in LB containing 320 mmol L^−1^ NaCl, by approximately 1.2‐ and 1.4‐fold, respectively, compared with *B. pseudomallei* cultured in 170 mmol L^−1^ NaCl at the 6‐hr time point (Pumirat et al., 2010). Indeed, the secretomic profile confirmed the presence of GroEL in the culture supernatant only after exposure to 320 mmol L^−1^ NaCl (Pumirat et al., 2009). Moreover, our results were consistent with the observation that inactivation of the *rpoE* operon increased susceptibility of *B. pseudomallei* to killing by menadione and H_2_O_2_ and high osmolarity (Korbsrisate et al., 2005). Furthermore, it has been demonstrated that *rpoE* regulated a heat‐inducible promoter of the *rpoH* gene in *B. pseudomallei* (Vanaporn et al., 2008). These data implied that RpoE plays an important role in the increased resistance of *B*. *pseudomallei* in response to heat and oxidative stress.

Among the salt‐altered genes of *B*. *pseudomallei* K96243 (Pumirat et al., 2010), we previously detected downregulation of the flagella biosynthesis sigma factor *fliA* gene (*bpsl3291*), by approximately 1.5‐ and 1.2‐fold (at 3 and 6 hr, respectively), when *B. pseudomallei* was grown in medium supplemented with 320 mmol L^−1^ NaCl compared with 170 mmol L^−1^ NaCl. This observation led us to examine whether growth of *B*. *pseudomallei* under high salt conditions affected the production of flagella. Under electron microscopic examination (Table 3), we found that most *B*. *pseudomallei* isolates grown under high salt conditions (300 mmol L^−1^ NaCl) did not produce flagella, whereas the majority of *B*. *pseudomallei* isolates grown under lower salt concentrations (0 and 150 mmol L^−1^ NaCl) presented at least one flagellum. The decreased expression of motility genes due to salt stress has also been documented for other bacteria such as *Sphingomonas* sp. strain LH128 (Fida et al., 2012) and *B. subtilis* (Hoper, Bernhardt, & Hecker, 2006; Steil, Hoffmann, Budde, Volker, & Bremer, 2003). All six *B*. *pseudomallei* isolates exhibited a smaller mean diameter for their motility zone when cultured under high salt conditions (300 mmol L^−1^ NaCl), compared with culturing under salt‐free or low salt conditions (0 and 150 mmol L^−1^ NaCl). This observation implied that salt stress plays an important role in regulating the production of bacterial flagella. One possible explanation for this is that in order to cope with stressful environmental conditions the bacteria conserve energy by diminishing nonvital activities, such as motility, by reducing the production of flagella by decreasing the expression of the motility regulator gene.

The ability to form a biofilm is important for *B. pseudomallei* to gain resistance to numerous environmental factors, including certain antibiotics and stresses (Cheng & Currie, 2005; Inglis & Sagripanti, 2006; Kamjumphol et al., 2013). Our study detected the increased ability of *B. pseudomallei* to form a biofilm when bacterial isolates were grown in medium supplemented with NaCl, compared with salt‐free medium (Table 4). This was consistent with the findings of Kamjumphol et al. who demonstrated that biofilm formation was increased when *B. pseudomallei* was grown in modified Vogel and Bonner's medium containing 0.85–1.7 mol L^−1^ NaCl (Kamjumphol et al., 2013). This indicated that *B. pseudomallei* responds to salt stress by producing a biofilm that could confer cross‐protection against other environmental stresses.

Exposure to high salinity is likely to be associated with pathogenesis in *B*. *pseudomallei*. Previously, invasion of A549 cells was enhanced by culturing of *B*. *pseudomallei* K96243 in salt‐supplemented LB medium (Pumirat et al., 2010). Our results showed that when grown in the presence of NaCl, all six *B*. *pseudomallei* isolates exhibited significantly increased plague formation in HeLa cells (Figure 4). The elevated rate of cellular invasion in response to NaCl may increase the load of intracellular bacteria, contributing to cell‐to‐cell spread or enhance cell cytotoxicity. Several studies have demonstrated the requirement of the Bsa T3SS and type VI secretion system (T6SS) for the intracellular pathogenicity of *B*. *pseudomallei* (Burtnick et al., 2008, 2011; Lim et al., 2015; Shalom, Shaw, & Thomas, 2007; Stevens et al., 2002; Warawa & Woods, 2005). We postulate that these systems may participate in the enhanced plaque formation of *B*. *pseudomallei* observed after exposure to NaCl. However, further experiments are required to investigate this possibility.

## 5 CONCLUSIONS

In conclusion, our results demonstrated that high salt conditions modulate adaptive responses in *B*. *pseudomallei* isolates. These adaptive responses include increased thermal resistance, plaque formation, and decreased flagella and oxidative susceptibility. Similar results were observed in all six isolates tested; suggesting that salt stress induces a general, conserved response in *B. pseudomallei*. Our findings provide insight into how these bacteria persist in endemic environments abundant in saline soil and water, and may indicate the link between the establishment and pathogenesis of *B*. *pseudomallei* infection in CF patients.

## CONFLICT OF INTEREST

The authors declare that they have no competing interests.
