# Supplementary figures and images for: Effects of sodium chloride on heat resistance, oxidative susceptibility, motility, biofilm and plaque formation of Burkholderia pseudomallei
Source: Microbiologyopen. 2017 Jun 23;6(4):e00493. doi: 10.1002/mbo3.493 (PMC5552950; doi:10.1002/mbo3.493)

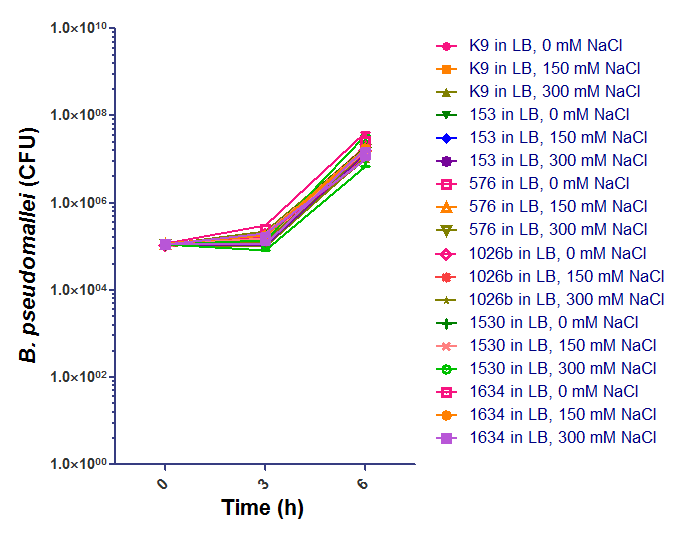

Supplement: Supplementary file 1 [file MBO3-6-na-s001.tif]

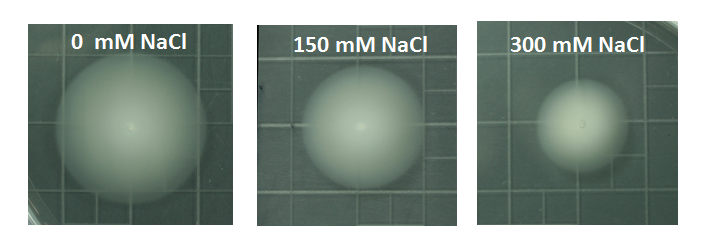

Supplement: Supplementary file 2 [file MBO3-6-na-s002.tif]

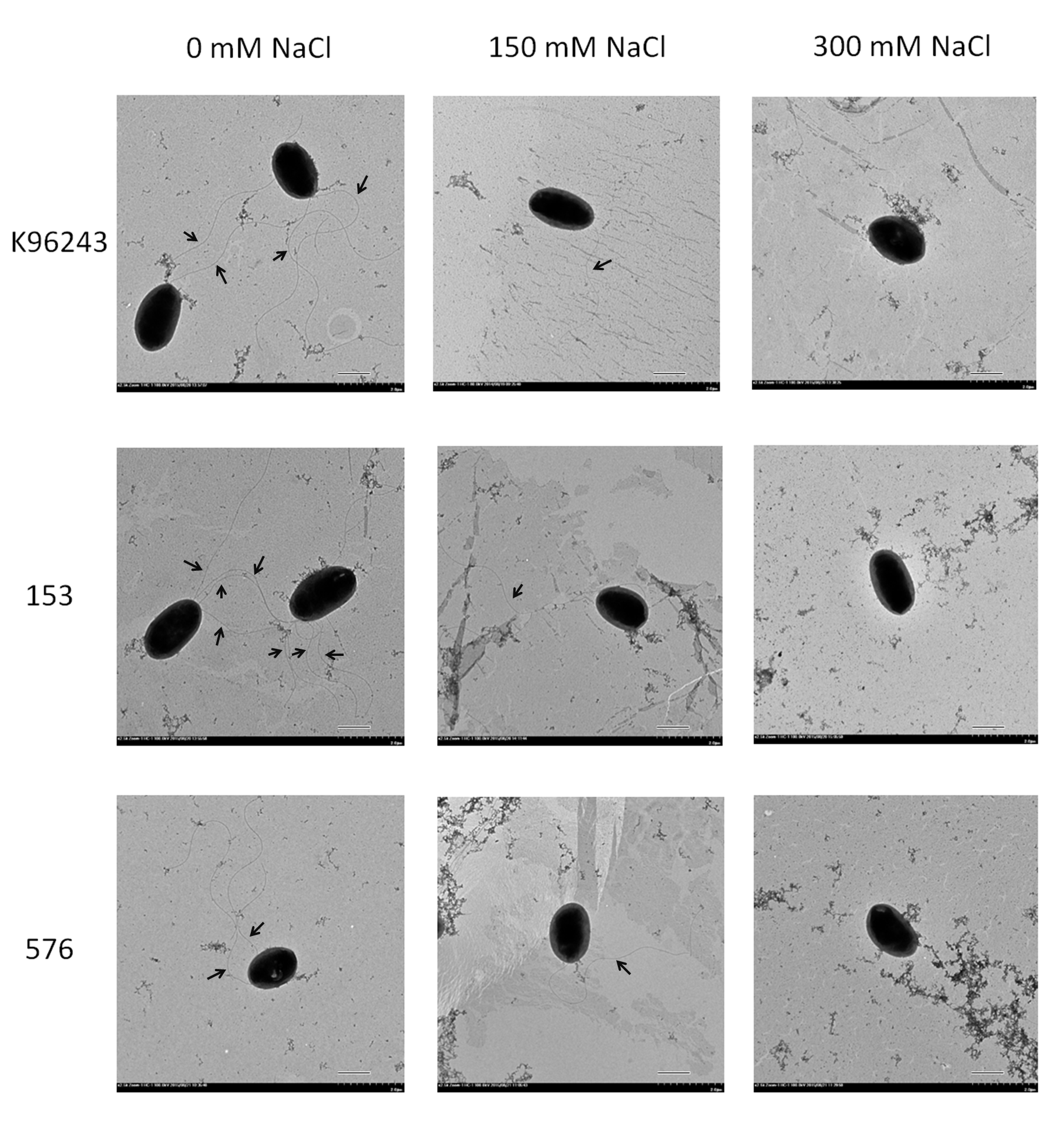

Supplement: Supplementary file 3 [file MBO3-6-na-s003.tif]
